# Evaluating Quality of Life of Prostate Cancer Patients After Radical Prostatectomy

**DOI:** 10.7759/cureus.79973

**Published:** 2025-03-03

**Authors:** Vissarion I Bakalis

**Affiliations:** 1 Department of Nursing, University of Thessaly, Larissa, GRC

**Keywords:** physical functioning, prostate cancer, quality of life (qol), radical prostatectomy, social function

## Abstract

Background

Prostate cancer is the second most commonly diagnosed cancer globally and has a significant impact on patients’ quality of life (QoL). The treatment of prostate cancer, particularly through radical prostatectomy, offers curative benefits but often results in adverse effects such as urinary incontinence and erectile dysfunction. These issues can persist long after treatment, affecting both physical and mental well-being. Beyond these complications, patients may also experience emotional distress, anxiety, and social implications, such as changes in relationships and self-perception. As such, evaluating QoL post-treatment is essential in understanding the comprehensive impact of prostate cancer care.

Methodology

This study involved 110 prostate cancer patients who underwent radical prostatectomy. QoL was assessed using the Short-Form Health Survey Questionnaire (SF-36) at the following three points: before treatment and at six and 12 months post-treatment. The evaluation focused on physical, emotional, and social aspects of health. Patients with psychiatric disorders or those unable to understand Greek were excluded from the study.

Results

The study participants’ average age ranged from 67 to 82 years (mean = 74.2 years). A significant improvement in social functioning was observed between the first and second assessments. Improvements were also seen in physical functionality, emotional well-being, and general health, but these changes were not statistically significant. By the third assessment (12 months post-surgery), significant improvements were noted in vitality, though other changes, including in physical, emotional, and role functioning, were not significant.

Conclusions

Radical prostatectomy is effective for cancer control but often results in persistent QoL issues such as erectile dysfunction and urinary incontinence. While there are improvements in certain areas such as vitality and social functioning, managing the full spectrum of physical and psychosocial impacts requires a holistic approach. Incorporating psychological support and sexual rehabilitation into treatment plans is essential to enhance the QoL of prostate cancer patients. The integration of patient-reported outcomes in clinical care is crucial for ensuring comprehensive and effective treatment.

## Introduction

Prostate cancer is the most prevalent non-cutaneous malignancy among men globally and the second most commonly diagnosed cancer, with an estimated 1,600,000 cases and 366,000 deaths annually. Due to the aging population, the incidence of prostate cancer is projected to increase to nearly 2.3 million new cases and 740,000 deaths by 2040 [1,2].

While prostate cancer treatment is advancing, it still frequently leads to adverse effects on patients’ quality of life (QoL) and urinary disease-specific symptoms [3]. As a result, prostate cancer survivors often experience long-term symptom burden that impact their perceived health status [4,5].

Radical prostatectomy has been shown to be an effective therapeutic intervention for patients with early-stage prostate cancer, conferring a well-documented long-term survival benefit [6]. The effects of radical prostatectomy extend beyond simple cancer control, including urinary incontinence and preservation of non-erectile function. Surgery is facilitated by advances in our understanding of prostate and cervical nerve anatomy, as well as the pioneering nerve-sparing radical prostatectomy technique developed by Walsh and Donker [7]. Recent studies have reported varying success rates of nerve-sparing techniques, with rates of recovery of erectile function ranging from 0% to expert factors and the extent of nerve preservation [6,7]. These findings underscore the continued improvements in surgical approaches to optimize functional outcomes. The recovery of urinary continence after radical prostatectomy is a crucial aspect for maintaining overall health and maximizing the outcomes of sexual rehabilitation. Following catheter removal, most patients experience some degree of urinary incontinence [8]. The incidence of post-prostatectomy urinary incontinence ranges from 0% to 87% [9].

The incidence of urinary incontinence varies based on the surgical approach and method but often improves within one to two years postoperatively. Studies have shown that persistent urinary incontinence affects approximately 5-15% of patients two years after radical prostatectomy, with variations depending on the surgical technique and patient-specific factors [10]. Nonetheless, a subset of patients continue to exhibit urinary incontinence. Urodynamic assessments have documented elevated rates of incontinence among individuals following radical prostatectomy [11].

Erectile dysfunction is a critical concern for the well-being of men treated for prostate cancer, as it is strongly linked to depression and significant emotional distress [12]. This condition is associated with depressive symptoms, even approximately four years post-diagnosis. These findings suggest that erectile dysfunction in men with prostate cancer can have enduring psychological effects [13]. Erectile dysfunction negatively impacts the QoL of men and their sexual partners, and the burden of erectile dysfunction often persists long after concerns about cancer cure have subsided [14].

A diagnosis of prostate cancer is associated with physical, psychological, emotional, social, and financial hardships and negatively impacts the QoL of men suffering from this condition [15,16]. These changes in QoL may significantly depend on factors directly linked to the tumor and/or treatment modalities [17,18]. For these reasons, assessing and prioritizing QoL is a vital consideration in cancer care and research. Increasingly, QoL metrics are being used as primary outcome measures to evaluate the efficacy of treatments and as endpoints for comparative analyses of different therapeutic approaches. Furthermore, the emerging diversity of treatment modalities and surgical techniques, coupled with the potential for combined interventions, necessitates a heightened focus on patients’ subjective sensations, expectations, and perceptions regarding the effects of treatment [19]. Incorporating patient-reported outcomes is crucial for evaluating the physical and psychosocial burdens associated with applied treatments, as well as for implementing targeted interventions that may enhance patients’ prospects and well-being. Therefore, validated multidimensional questionnaires with standardized methodologies, which can enable researchers and clinicians to assess the impact on QoL, should be widely adopted [19,20].

This prospective study investigates alterations in health-related QoL among prostate cancer patients before and after undergoing radical prostatectomy. Additionally, the study aims to identify the specific aspects of prostate cancer patients’ lives that are most significantly impacted by radical prostatectomy.

## Materials and methods

In this study, a total of 110 patients who were recently diagnosed with prostate cancer (prospective study) and had received surgical treatment were recruited. The evaluation was performed before treatment and six and 12 months after treatment. The study excluded patients who could not understand the Greek language as well as those with psychiatric disorders owing to insufficient valid data of these social groups or to exclude factors not related to the present study. A prerequisite for study participation was patient consent, which was also granted in writing by the participants.

The QoL was assessed using the Short-Form Health Survey Questionnaire (SF-36) [21]. The SF-36 is a generic multidimensional measurement instrument comprising 36 items across the following eight domains: physical functioning, role limitations due to physical health, bodily pain, general health perceptions, energy/fatigue, social functioning, role limitations due to emotional problems, and emotional well-being. The final score ranged from 0 to 100, with 0 indicating the poorest health state and 100 representing the optimal health state. The Cronbach’s alpha coefficient of the total SF-36 was 0.87, and for the eight subscales ranged from 0.85 to 0.87, all above the recommended value of 0.7 [22].

Sample selection

The study sample was recruited using convenience sampling conducted at the Urology Clinic of the University Hospital of Larissa from February 2019 to February 2020. The Ethics Committee of the Faculty of Medicine of the University of Thessaly (approval number: 1/11-02-2019) reviewed and approved the study, and all participants provided informed consent.

The study sample was selected from newly admitted patients with a recent diagnosis of prostate cancer attending the hospital for initiation of surgical treatment. The researcher, in collaboration with the medical and nursing staff of the clinic, was informed daily about the admissions to identify patients who met the study criteria. The patient was then approached and asked for their consent to participate in the study after being verbally informed of the purpose of the study, the anonymity and confidentiality of the data, and the possibility of withdrawing from the study at any time.

After consent, patients answered the self-completed questionnaires on the same day. The questionnaire was completed at three different time intervals, i.e., before surgery, six months after surgery, and 12 months after surgery.

Statistical analysis

SPSS version 25.0 (IBM Corp., Armonk, NY, USA) was used for statistical analysis. Non-parametric methods were used to compare the scores of the QoL assessment tools between the patients’ and their partners’ assessments. Wilcoxon test was used to compare pairwise non-normal distributions, patients’ ratings on each of the three study measures, and patients’ ratings on each of the study measures. Statistical significance was set at 0.05.

## Results

The age of study population ranged from 67 to 82 years (mean ± SD = 74.20 ± 4.42 years). Patients did not observe a significant change in their functional ability from the first to the second measurement using the SF-36 questionnaire. Statistically significant differences were only found in patients’ assessments regarding social functioning (p = 0.035). Patients described a significant improvement in social functioning on the second measurement (mean ± SD = 52.0 ± 24.2 vs. 45.0 ± 24.8) (Table 1).

**Table 1:** Mean values, SDs, and comparison of patients’ assessment of functional ability at the first and second measurement using the SF-36 questionnaire. SF-36 = Short-Form Health Survey Questionnaire

	First measurement (before treatment)	Second measurement (6 months after treatment)		
Functional capacity subscale (0–100)	Mean ± SD	Mean ± SD	Mean ± SD	P-value (Wilcoxon)
Physical functioning	50.3 ± 12.8	49.6 ± 12.1	0.6 ± 18.7	0.604
Role limitations due to physical health	53.0 ± 24.9	50.5 ± 26.3	2.5 ± 35.7	0.362
Role limitations due to emotional problems	53.0 ± 32.3	51.2 ± 28.5	1.7 ± 40.3	0.509
Energy/Fatigue	48.5 ± 16.4	49.9 ± 18.6	-1.3 ± 25.1	0.490
Emotional well-being	50.1 ± 15.9	51.0 ± 15.5	-0.8 ± 23.5	0.858
Social functioning	45.0 ± 24.8	52.0 ± 24.2	-7.0 ± 35.7	0.035
Pain	48.7 ± 22.6	46.6 ± 23.2	2.0 ± 30.8	0.380
General health	48.2 ± 16.6	50.1 ± 16.1	-1.9 ± 23.1	0.420
Health change	47.5 ± 37.0	49.8 ± 36.3	-2.3 ± 52.9	0.602

In addition, patients described improvements in their physical functionality (mean ± SD = 49.6± 12.1 vs. 50.3 ± 12.8), role limitations due to physical health (mean ± SD = 50.5 ± 26.3 vs. 53 ± 24.9), and role limitations due to emotional problems (mean ± SD = 51.2 ± 28.5 vs. 53 ± 32.3). They also reported improvement in their physical pain (mean ± SD = 46.6 ± 23.2 vs. 48.7 ± 22.6) and general health (mean ± SD = 50.1 ± 16.1 vs. 48.2 ± 16.6). In addition, they observed a change in their health compared to one year ago (mean ± SD = 49.8 ± 36.3 vs. 47.5 ± 37).

Patients reported deterioration in social functioning, emotional happiness, and role functioning due to emotional problems, as measured by the SF-36 questionnaire between the second and third assessments. Additionally, they experienced an improvement in their vitality. However, none of these differences were statistically significant (Table 2).

**Table 2:** Mean values, standard deviations, and comparison of patients’ functional assessment between the second and third measurement using the SF-36 questionnaire. SF-36 = Short-Form Health Survey Questionnaire

	Second measurement (6 months after treatment)	Third measurement (12 months after treatment)		
Functional capacity subscale (0–100)	Mean ± SD	Mean ± SD	Mean ± SD	P-value (Wilcoxon)
Physical functioning	49.6 ± 12.1	49.0 ± 13.0	0.6 ± 17.5	0.692
Role limitations due to physical health	50.5 ± 26.3	51.5 ± 24.0	-0.9 ± 33.4	0.799
Role limitations due to emotional problems	51.2 ± 28.5	49.7 ± 30.0	1.5 ± 39.5	0.611
Energy/Fatigue	49.9 ± 18.6	53.3 ± 17.8	-3.4 ± 23.6	0.062
Emotional well-being	51.0 ± 15.5	47.6 ± 16.2	3.3 ± 23.2	0.066
Social functioning	52.0 ± 24.2	50.3 ± 24.6	1.6 ± 34.8	0.729
Pain	46.6 ± 23.2	46.3 ± 20.3	0.3 ± 30.3	0.949
General health	50.1 ± 16.1	51.8 ± 16.1	-1.6 ± 21.3	0.363
Health change	49.8 ± 36.3	49.6 ± 36.4	0.1 ± 54.5	0.979

Patients who completed the study described deterioration in physical, emotional, and role functioning. In contrast, they described improvement in their vitality, social functioning, and general health with the SF-36 questionnaire, of which only vitality was found to be statistically significant (p = 0.013). The other observed differences between patients’ responses did not differ statistically significantly (p > 0.050) (Table 3).

**Table 3:** Mean values, standard deviations, and comparison of patients’ functional assessment between the first and third measurement using the SF-36 questionnaire. SF-36 = Short-Form Health Survey Questionnaire

	First measurement (before treatment)	Third measurement (12 months after treatment)		
Functional capacity subscale (0–100)	Mean ± SD	Mean ± SD	Mean ± SD	P-value (Wilcoxon)
Physical functioning	50.3 ± 12.8	49.0 ± 13.0	1.3 ± 18.2	0.479
Role limitations due to physical health	53.0 ± 24.9	51.5 ± 24.0	1.5 ± 34.2	0.643
Role limitations due to emotional problems	53.0 ± 32.3	49.7 ± 30.0	3.3 ± 41.6	0.326
Energy/Fatigue	48.5 ± 16.4	53.3 ± 17.8	-4.8 ± 23.4	0.013
Emotional well-being	50.1 ± 15.9	47.6 ± 16.2	2.5 ± 2.0	0.344
Social functioning	45.0 ± 24.8	50.3 ± 24.6	-5.3 ± 36.3	0.141
Pain	48.7 ± 22.6	46.3 ± 20.3	2.4 ± 29.8	0.384
General health	48.2 ± 16.6	51.8 ± 16.1	-3.6 ± 23.0	0.104
Health change	47.5 ± 37.0	49.6 ± 36.4	-2.1 ± 50.6	0.674

## Discussion

This prospective study aimed to investigate changes in health-related QoL among prostate cancer patients before and after undergoing radical prostatectomy. Specifically, the study aimed to identify which aspects of the prostate cancer patients’ lives are most significantly impacted by the surgical procedure. The study included male patients aged 67 to 82 years, with a mean age of 74.20 ± 4.42 years. Key findings from the study indicate that patients did not observe a significant change in their functional ability between the first and second measurements, as assessed using the SF-36 questionnaire. Statistically significant differences were only found in patients’ assessments regarding social functioning, with a notable improvement observed in the second measurement (p = 0.035). Specifically, the mean score for social functioning improved from 45.0 ± 24.8 to 52.0 ± 24.2. The concept of QoL, particularly health-related QoL, encompasses the physical, psychological, and social domains, which are viewed as distinct yet interrelated areas, and are influenced by an individual’s beliefs, attitudes, values, and perception of their health status [23].

Our findings reveal substantial improvements across multiple domains, including physical, emotional, and cognitive functioning, as well as a marked enhancement in the overall QoL experienced by our participants over the one-year study period. Notably, these improvements were sustained without any observed deterioration even at the six-month interim assessment, underscoring the enduring benefits of the intervention. Importantly, our results indicate no detrimental impact on functional scales following radical prostatectomy, which aligns with the findings of a previous German study utilizing the European Organisation for Research and Treatment of Cancer Quality of Life Questionnaire Core 30 and European Organization for Research and Treatment of Cancer Quality of Life Questionnaire-Prostate instruments and a US study employing the SF-36 and University of California, Los Angeles questionnaires [24]. For instance, a longitudinal investigation conducted by Litwin et al. [24] found that over 90% of radical prostatectomy patients attained their baseline health-related QoL status at the one-year postoperative mark. Our findings similarly demonstrate that several symptom domains, including pain, energy, and general health, experienced significant improvements over time following radical prostatectomy. The observation that numerous health-related QoL dimensions at the one-year postoperative stage were markedly elevated compared to baseline suggests that the impaired baseline QoL in patients with untreated prostate cancer was subsequently ameliorated over time after undergoing surgical treatment, aligning with the conclusions of a previous study [24].

Erectile function recovery after radical prostatectomy varies significantly based on factors such as differences in erectile dysfunction definition, data collection methods, post-surgical time point, and population studied. However, most studies have not focused on the couple for interventions and evaluations. While the potential benefits of assessing the patient’s partner remain to be fully explored due to the scarcity of this methodological approach, psychotherapy has been shown to improve outcomes in this context [25].

Erectile dysfunction is a critical and often debilitating issue that significantly impairs the QoL of men treated for prostate cancer. This condition is strongly associated with increased rates of depression and significant psychological distress, which can further exacerbate the immense challenges and struggles faced by these vulnerable patients. The inability to engage in intimate relationships and the loss of sexual function can lead to feelings of inadequacy, lowered self-esteem, and a profound sense of isolation, all of which can severely impact the overall well-being and QoL of these individuals. Addressing this complex issue requires a multifaceted approach, encompassing both medical interventions and psychological support, to provide comprehensive care and help these patients overcome the profound impact of erectile dysfunction on their lives [13,26,27].

Individuals with greater impairment of physical and mental wellbeing are less likely to achieve successful sexual rehabilitation following treatment. Distress regarding sexual dysfunction negatively impacts their perceptions of their current physical and mental health. Factors beyond the effects of age, health, and treatment that are strongly associated with long-term sexual satisfaction are more cognitive and behavioral, for instance, having one or more sexually functional partners, having experienced good sexual function before treatment, and prioritizing the preservation of sexual function when selecting a treatment. These factors may be targeted by sexual counseling interventions to complement and enhance efforts during medical treatment for individuals with erectile dysfunction [28]. The masculine identity of men is significantly influenced by their experiences of prostate cancer and the consequences of surgical treatment. The loss of erectile function, a common side effect of prostate cancer treatment, has a substantial impact on men’s perception of masculinity. While men often rely on stereotypically masculine qualities, such as emotional control and rationality, when describing their reactions to the diagnosis and treatment of cancer, they also grapple with a newfound sense of physical vulnerability. Gannon et al. suggested that addressing these issues may involve a careful examination of the language employed when discussing health, illness, and gender [29].

A key component is the educational and informative aspect of the disease to address misconceptions and dispel myths, creating space for expression and emotional connection. Identifying the patient’s personal needs, supporting their self-management to improve their QoL within their constraints, and, importantly, their capacity to serve as an information resource for their social network, are fundamental and emphasized throughout the treatment process. Adopting an integrated approach, where psychological and sexual therapies hold equal significance, we must comprehend, support, and direct patients, their partners, and their family members.

Study limitations

This study was limited by the use of a non-validated questionnaire as well as response bias because respondents may have been more interested in participating than non-respondents. In addition, the results of this study should be interpreted with caution. The sample of patients and by extension their partners was recruited through convenience sampling rather than random sampling. Although the findings possibly lead to the conclusion that the QoL of the patients who completed the study changed, they cannot be generalized to the entire population.

## Conclusions

Prostate cancer remains a major health issue globally, with significant impacts on patients’ physical, emotional, and psychological well-being. The findings of this study reveal that while radical prostatectomy can provide effective cancer control and improvements in certain aspects of QoL, challenges such as erectile dysfunction and urinary incontinence continue to affect patients post-surgery. Notably, improvements in vitality and social functioning were observed, although these were not universally significant. This highlights the complexity of managing post-treatment QoL, where both physical recovery and psychosocial aspects play crucial roles. The integration of psychological support and sexual rehabilitation into prostate cancer care is essential for addressing the broader needs of patients and enhancing their QoL. As prostate cancer treatment evolves, patient-reported outcomes must continue to inform clinical practices, ensuring a holistic approach to care that recognizes the multifaceted consequences of the disease and its treatment.
